# Evaluation of the Effect of Proptosis on Choroidal Thickness in Graves’ Ophthalmopathy

**DOI:** 10.4274/tjo.galenos.2020.97415

**Published:** 2020-08-26

**Authors:** Gamze Yıldırım, Esra Şahlı, Mehmet Numan Alp

**Affiliations:** 1 Niğde Ömer Halisdemir Training and Research Hospital, Clinic of Ophthalmology, Niğde, Turkey; 2 Ankara University Faculty of Medicine, Department of Ophthalmology, Ankara, Turkey; 3 Ankara Numune Training and Research Hospital, Clinic of Ophthalmology, Ankara, Turkey

**Keywords:** Graves’ ophthalmopathy, choroidal thickness, optical coherence tomography, proptosis

## Abstract

**Objectives:**

To evaluate the effect of proptosis on choroidal thickness in patients with Graves’ ophthalmopathy.

**Materials and Methods:**

Twenty-five eyes of 25 Graves’ patients with proptosis, 25 eyes of 25 Graves’ patients without proptosis, and 25 eyes of 25 healthy individuals were included in this prospective study. The subfoveal choroidal thickness and choroidal thicknesses at 6 points from the fovea at 500 μm intervals were measured by Cirrus HD-OCT. All measurements were compared among the proptosis, non-proptosis, and control groups and the active, inactive, and control groups.

**Results:**

The mean subfoveal choroidal thickness in the proptosis group was 289.7±68.5 μm, 322.5±55.8 μm in the non-proptosis group, and 316.1±63.0 μm in the control group. The mean nasal choroidal thickness was 260.5±63.5 μm in the proptosis group, 293.9±57.9 μm in the non-proptosis group, and 279.5±63.1 μm in the control group. The mean temporal choroidal thickness was 261.8±60.9 μm in the proptosis group, 289.0±51.8 μm in the non-proptosis group, and 287.8±56.2 μm in the control group. Mean choroidal thickness was 264.7±58.5 μm in the proptosis group, 296.2±47.5 μm in the non-proptosis group, and 288.3±55.1 μm in the control group. There were no statistically significant differences among the groups with respect to choroidal thickness measurements (p>0.05).

**Conclusion:**

No significant difference in choroidal thickness was detected between Graves’ patients with and without proptosis and the controls. There was no effect of clinical activation on choroidal thickness.

## Introduction

Graves’ ophthalmopathy is an ocular condition that frequently manifests with thyroid dysfunction. It is usually seen in patients with Graves’ disease.^1^ Graves’ disease is characterized by hyperthyroidism, diffuse goiter, ophthalmopathy, and in rare cases, dermopathy. In addition to elevated free thyroid hormone and suppressed thyroid stimulating hormone (TSH) levels, serum antithyroglobulin antibodies, antithyroid peroxidase (anti-TPO), and TSH receptor antibodies may also be detected in Graves’ disease.^2^

While Graves’ ophthalmopathy is 2.5 to 6 times more common among women, severe disease is more common among men. The onset of the disease is generally between the ages of 30 and 50, and the course becomes more severe after the age of 50.^3^ Ophthalmopathy is the most common extrathyroidal finding, present in 25 to 50% of the patients with Graves’ disease. While subclinical involvement is present in most cases, only about 3 to 5% of patients with Graves’ ophthalmopathy have severe ophthalmopathy.^3^ The main clinical manifestations of the disease can be summarized as periorbital edema, eyelid retraction, proptosis, restrictive myopathy, and optic neuropathy. Proptosis results from inflammation and increased volume of orbital soft tissues, including the extraocular muscles.^4^

Although the pathogenesis of the disease is not completely understood, it has been established that the autoimmunity developed against the antigens common to the thyroid gland and the orbit plays the main role. Reactive T-lymphocytes that recognize these antigens infiltrate the orbit and extraocular muscles. Cytokines secreted by T-lymphocytes stimulate the synthesis and secretion of glycosaminoglycans (GAG) by fibroblasts, which leads to expansion of the orbital contents.^5^ As the orbital pressure increases, venous circulatory disturbance and venous stasis may occur.^6^

The choroid is composed of a vascular network, which acts as a blood supply for the outer retina, optic nerve, and avascular fovea. The choroid tissue is capable of rapidly changing its thickness in response to various stimuli. Changes in the choroidal circulation, autonomic nervous system inputs, or inflammation may lead to a change in choroidal thickness.^7^ A relatively new technique, enhanced depth imaging optical coherence tomography (EDI-OCT) using longer wavelength light, allows for rapid and precise measurement of choroidal thickness. The aim of this study was to evaluate the effect of proptosis on choroidal thickness in patients with Graves’ ophthalmopathy.

## Materials and Methods

This prospective trial was performed at the Clinic of Ophthalmology, Ankara Numune Training and Research Hospital, between 2014 and 2016. A total of 50 consecutive patients with a diagnosis of Graves’ disease (25 patients with proptosis and 25 patients without proptosis) and 25 healthy control subjects were studied. The study protocol was approved by the local ethics committee (17.07.2014, decision number: 14-257). Written informed consent was obtained from all patients and controls. The study was conducted in accordance with the principles of the Declaration of Helsinki.

Patients with myopic, hyperopic, or astigmatic refractive errors greater than 3.0 diopters (D); patients with ocular disease such as uveitis, retinal and choroidal diseases, optic nerve pathologies, strabismus, or amblyopia; and patients with a history of ocular trauma or surgery were excluded from the study. Patients who were under 18, pregnant, or breastfeeding were also excluded. Age and gender-matched healthy controls were selected from the outpatient facility of the department.

A detailed medical history was obtained from all the patients and controls. Each subject underwent a complete ophthalmic examination including best corrected visual acuity, slit lamp biomicroscopy, intraocular pressure (IOP) measurements in primary gaze position and upgaze with non-contact tonometry, fundus evaluation after pupil dilatation with the slit lamp using a 90.0 D lens, color vision, direct-indirect pupillary reactions, and eye movements. IOP readings were adjusted according to central corneal thickness. IOP and corneal thickness measurements were obtained by a fully automatic non-contact tonometer combined with a pachymeter, Canon TX-20P (Canon Medical Systems Europe B.V., Zoetermeer, the Netherlands). Axial length was measured by ultrasound, Aviso A/B scan, and ultrasound biomicroscopy (UBM) (Quantel Medical, Cournon d’Auvergne Cedex, France). The amount of proptosis was measured using a Hertel exophthalmometer by a single physician (G.Y.) in each case. Computerized tomography (CT) of the orbit was performed to evaluate involvement of the extraocular muscles in all patients with Graves’ disease. Extraocular muscle enlargement and apical crowding signs in CT imaging were accepted as extraocular muscle involvement.

Only patients with Hertel exophthalmometer readings greater than 20 mm, a difference in readings of 2 mm or more between the two eyes, and reported proptosis on orbital CT by a radiologist were included in the proptosis group. The same patients who were measured as proptotic with the Hertel exophthalmometer were also reported as proptotic in the orbital CT. Measurement of proptosis with CT was calculated by measuring the perpendicular distance from the posterior globe margin to the interzygomatic line on axial scans at the midglobe level. The distance from the posterior sclera margin to the interzygomatic line is normally 9.9±1.7 mm.^8^

All patients were assessed using the European Group on Graves’ Orbitopathy (EUGOGO) staging and Clinical Activity score (CAS) to define the stage and the activity of ophthalmopathy.^9,10^ As recommended by EUGOGO, patients meeting at least one of the following criteria were classified as having mild disease: minor lid retraction (less than 2 mm), mild soft tissue involvement, exophthalmos less than 3 mm, transient or no diplopia, and corneal exposure responsive to lubricants. Patients with lid retraction equal or greater than 2 mm, moderate or severe soft tissue involvement, exophthalmos equal or greater than 3 mm, or inconstant or constant diplopia were classified as having moderate to severe disease. Patients with optic neuropathy and/or corneal breakdown were classified as having sight-threatening disease.^9^ CAS was assessed by scoring 1 point for each sign present (spontaneous orbital pain, gaze-evoked orbital pain, eyelid swelling, eyelid erythema, conjunctival redness, chemosis, and caruncle/plica inflammation). Active disease was defined as CAS of 3 and above, inactive disease as CAS below 3.^10^

The current medical therapy status of patients was categorized as no medical treatment, antithyroid drug therapy, and combination therapy with anti-thyroid drugs and β-blocker drugs.

One eye per patient was selected and examined. For each patient in the proptosis group, the eye with the higher Hertel exophthalmometry measurement and in each patient in the non-proptosis group, the eye at the higher stage in EUGOGO system was examined for the purpose of the study. If both the eyes of the patient behaved similarly, the eye with the higher value of signal strength on the OCT scan was chosen to study. For each control subject, the eye with the higher value of signal strength on the OCT scan was included. If the signal strengths of both eyes were equal, a simple random selection was done.

All of the subjects were scanned with the Cirrus HD-OCT (Carl Zeiss Meditec Inc., Dublin, CA). The HD 5 Line Raster scan protocol spaced at 0.25 mm was performed centering on the fovea and consisted of 5 parallel lines of 1024 A-scans per B-scan. The HD line scans were performed in EDI-OCT mode. Choroidal thickness was measured manually as the vertical distance between the outer border of the hyperreflective line corresponding to the retinal pigment epithelium and the inner surface of the sclera. The subfoveal choroidal thickness and choroidal thicknesses at points 500 µm, 1000 µm, and 1500 µm temporal and nasal to the fovea were measured simultaneously. Images with signal strength equal to or greater than 6 were included in the study. The subfoveal measurement, the average of the 3 temporal measurements, the average of the 3 nasal measurements, and the average of all 7 measurements (mean choroidal thickness) were used for the statistical analysis.

### Statistical Analysis

Statistical analysis was performed using SPSS 22.0 for Windows. The results were reported as mean ± standard deviation. The chi-square test was used to examine differences among categorical data. The variables were tested for normality and homogeneity by using the Kolmogorov-Smirnov and Levene test. The intergroup differences in numerical variables were analyzed by independent-samples t-test and Mann-Whitney U test. Univariate analysis and Tukey HSD tests were used for data analysis. Correlations between the variables were tested with Pearson and Spearman correlation coefficients. Multiple linear regression analyses were applied to reveal the predictors of choroidal thickness. P<0.05 was considered statistically significant.

## Results

Twenty-five Graves’ patients (5 men, 20 women) with proptosis, 25 Graves’ patients (8 men, 17 women) without proptosis, and 25 healthy subjects (9 men, 16 women) were included in the study. There was no statistically significant difference in sex among the groups. The mean age of the proptosis group, the non-proptosis group, and the control group was 42.2±10.8 years, 36.4±10.4 years, and 40.2±10.9 years, respectively. There was no statistically significant difference in mean age among the three groups. The mean duration of the disease was 24 months (ranging between 1 and 360 months) in the proptosis group and 6 months (ranging between 1 and 262 months) in the non-proptosis group. The number of patients with hyperthyroidism was 12 (48%) and 13 (52%) in the proptosis and non-proptosis groups, respectively. History of radioactive iodine (RAI) therapy was present in 4 patients (16%) in the proptosis group and 1 patient (4%) in the non-proptosis group. No significant difference was found between the 2 patient groups in disease duration, prevalence of hyperthyroidism, or history of RAI therapy. The numbers of current smokers were statistically similar in all 3 groups (data not shown). There was no statistically significant difference between the groups in terms of medical treatment status (data not shown). Clinical findings of the patients in the proptosis group and non-proptosis group were summarized in Table 1.

The mean axial lengths of the patients in the proptosis group, non-proptosis group, and control subjects were 23.1±0.9 mm, 23.0±0.8 mm, and 23.7±0.9 mm, respectively. A statistically significant difference was found between the non-proptosis group and the control group (p=0.016).

The average Hertel exophthalmometric values were 22.6±1.8 mm (19-27) in the proptosis group, 16.0±2.2 mm (14-20) in the non-proptosis group, and 15.0±1.2 mm (14-18) in the control group (p<0.001). The corrected IOP measurements in primary gaze position and upgaze were compared among the 3 groups (Table 2). There was no statistically significant difference among the groups in the corrected IOP measurements in primary gaze position. The corrected IOP values in upgaze were significantly higher in the proptosis group than the other groups (p=0.003).

Subfoveal choroidal thickness, average nasal choroidal thickness, average temporal choroidal thickness, and mean choroidal thickness values were given in Table 3. There were no statistically significant differences among the groups with respect to choroidal thickness measurements.

There were 9 patients (36%) with active disease (CAS of 3 or higher) in the proptosis group. None of the patients in the non-proptosis group were evaluated as clinically active. Based on the disease severity classification (EUGOGO), 12 patients (48%) in the proptosis group had mild ophthalmopathy, 10 patients (40%) had moderate to severe involvement, and 3 patients (12%) had sight-threatening disease. All of the patients in the non-proptosis group were classified as mild in severity.

After we classified patients as clinically active (n=9), inactive (n=41), and controls (n=25) according to CAS, we further evaluated the distribution of demographic data, IOP measurements, and axial length value in the active, inactive, and control groups. There were no statistically significant differences among the groups with respect to age, sex, disease duration, or number of current smokers. The corrected IOP measurements in primary gaze position and upgaze were statistically similar among the active, inactive, and control groups. The mean axial lengths of the patients were 23.5±0.9 mm in the active group, 22.9±0.8 mm in the inactive group, and 23.7±0.9 mm in controls. The mean axial length was significantly shorter in the inactive group (p=0.003).

We compared the active, inactive and control groups with respect to subfoveal choroidal thickness, average nasal choroidal thickness, average temporal choroidal thickness, and mean choroidal thickness values. All of the choroidal thickness measurements were found to be statistically thinner in the active group than in the inactive and control groups (Table 4).

According to the multiple linear regression analysis, disease activity was no longer significantly associated with choroidal thickness (p values for subfoveal, nasal, temporal and mean choroidal thicknesses were 0.725, 0.712, 0.772, and 0.737, respectively). Age and axial length were identified as independent risk factors for choroidal thickness.

In addition, we did not classify according to muscle involvement, but there was no significant difference between the control group and the EUGOGO mild, moderate, and severe groups in terms of subfoveal, nasal, temporal, and mean choroidal thicknesses (p=0.198; p=0.325; p=0.264; p=0.229).

## Discussion

Graves’ ophthalmopathy is characterized by autoimmune-induced inflammation of the extraocular muscles and orbital soft tissue. It has been demonstrated that infiltration of extraocular muscles and orbital adipose tissue by GAG, lymphocytes, and immune complexes lead to edema, soft tissue expansion, increased intraorbital volume, and subsequently increased intraorbital pressure.^11^

Increased intraorbital pressure may affect hemodynamics and can lead to inadequate venous circulation and a decrease in superior ophthalmic venous blood flow.^6,12,13^ Alp et al.^6^ demonstrated increased orbital venous congestion and decreased flow velocity in the superior ophthalmic vein in patients with ophthalmopathy compared to both patients with Graves’ disease without ophthalmopathy and healthy controls. Reversed flow in the superior ophthalmic vein was documented in 13% of patients with Graves’ ophthalmopathy. In a recent study, Nik et al.^14^ reported a significant delay in choroidal filling and loss of details in the choroidal vasculature on indocyanine green angiography in Graves’ ophthalmopathy patients with compressive optic neuropathy. The study explained that orbital inflammation and increased intraorbital pressure could be a possible pathogenic mechanism for the development of these abnormal choroidal filling patterns.

Few studies have assessed choroidal thickness in thyroid patients. Ulaş et al.^15^ found that the choroidal thickness was greater in all subgroups of hypothyroid patients (subclinical hypothyroid patients, overt hypothyroid patients, and euthyroid patients receiving levothyroxine treatment) compared to healthy subjects. They reported that GAG production and accumulation in the connective tissue could be responsible for the increase in choroidal thickness.

In a study of 62 eyes of 31 patients with Graves’ ophthalmopathy, the subfoveal choroid was found to be thicker in patients with ophthalmopathy than healthy controls, and choroidal thickness was correlated with CAS. The authors reported that the thicker choroid may be caused by venous obstruction and orbital congestion in these patients.^16^ Çalışkan  et al.^17^ compared subfoveal choroidal thickness in patients with active and inactive Graves’ ophthalmopathy patients to that in control subjects. The mean subfoveal choroidal thickness was found to be significantly greater in patients with active disease than in patients with inactive disease and controls. They also showed a positive correlation between CAS and subfoveal choroidal thickness. The increase in choroidal thickness in patients with active ophthalmopathy was thought to be associated with decreased orbital venous drainage due to vascular compression in the orbital space. Bruscolini et al.^18^ also observed higher subfoveal choroidal thickness values in Graves’ ophthalmopathy patients and reported a significant direct correlation between choroidal thickness and CAS score as well as with proptosis level measured by Hertel exophthalmometry. In a similar study, Cagiltay et al.^19^ included euthyroid patients in the study to eliminate the effect of hyperthyroidism on choroidal circulation and evaluated disease activity with vision, inflammation, strabismus, and appearance (VISA) score. Mean, subfoveal, and temporal choroidal thicknesses were higher in the study group and mean choroidal thickness was positively correlated with VISA score.^19^ In accordance with these studies, Yu et al.^20^ found that choroidal thickness was significantly increased in Graves’ ophthalmopathy patients. However, they could not demonstrate any relationship between choroidal thickness and CAS, degree of proptosis, thyroid function tests, or TSH receptor antibody levels. In a very recent study, subfoveal and temporal choroidal thickness values were significantly higher in the active ophthalmopathy group than in the stable group.^21^ The authors attributed the higher choroidal thickness in the active group to increased intraorbital pressure leading to impaired venous drainage and inflammation. They did not report a correlation between choroidal thickness and CAS due to the small number of patients and the lack of equal distribution of activity scores.^21^

Contrary to these studies, we found no statistically significant difference in choroidal thickness among the proptosis group, non-proptosis group, and the controls. These results show that the correlation between blood flow and choroidal thickness is not very obvious. We need to be able to explain why patients did not have thicker choroid than controls, as we expected. There is evidence indicating that non-vascular smooth muscle in the choroid is controlled by both sympathetic and parasympathetic systems. These autonomic inputs might make the muscles contract, eject fluid from the choroid, and make the choroid thinner.^7,22^ Although the role of the autonomic nervous system in the regulation of choroidal thickness is not well understood, the choroid might also be affected by increased sympathetic activity in Graves’ disease. In previous studies, it was suggested that inflammation may be the cause of choroidal thickening. It is also known that the choriocapillaris and choroidal vascular layers are fibroblast-rich tissue.^22^ Inflammation may cause fibrosis and thinning of choroid tissue.

As opposed to other studies done in this field, we found that the choroid was significantly thinner in patients with active disease compared to patients with inactive disease and the control group. When CAS was evaluated as an independent risk factor in choroidal thickness by multiple regression analysis, the CAS lost its significance as an independent risk factor (p=0.737); choroidal thickness was found to be related to axial length (p=0.001) and age (p=0.004). This may be due to the fact that our cases were not grouped according to CAS. Our study groups were mainly designed according to the presence of proptosis. There were only 9 patients in the active group, but 41 patients in the inactive group. Due to the lack of equal distribution in the groups, this result may not be included. Further studies that have a similar number of patients in the active proptosis group and the inactive proptosis group are needed.

There are many studies in the literature assessing the effect of age, sex, axial length, smoking, and diurnal rhythm on choroidal thickness.^23,24,25,26,27,28,29^ Fujiwara et al.^30^ showed that subfoveal choroidal thickness in the Japanese population decreased by 20 µm every 10 years. Another study showed that choroidal thickness was significantly greater in males than in females.^31^ Li et al.^24^ found a reduction of 58.2 µm in subfoveal choroidal thickness per 1 mm increase in axial length. Similar to these studies, only age and axial length were determined as independent risk factors for choroidal thickness in our study. In a study evaluating the diurnal change in choroidal thickness, the choroid was found to be thinner in the morning and thickest at night.^32^ Our measurements were performed between 10 am and 3 pm.

Increased episcleral venous pressure has been shown to be the most important cause of IOP increase in patients with Graves’ ophthalmopathy.^33^ No glaucomatous patients were included in our study and there was no difference in corrected IOP between the proptosis and non-proptosis and between the clinically active and inactive groups. In our study, the corrected IOP values in upgaze measured in the proptosis group were significantly higher than in the non-proptosis and control groups. This increase in IOP in the proptosis group may be due to the globe pressure caused by fibrotic and tense extraocular muscles.

The absence of choroidal auto-segmentation in the current OCT device is a limiting factor in our study. In two studies in the literature about choroidal thickness in Graves’ ophthalmopathy, both eyes of each patient were included in the study, whereas we included only one eye of each patient in our analysis. This approach was planned based on the possibility that two measurements taken from a single patient may negatively affect the statistical value.

Another limitation of our study is that we did not evaluate the correlation between CAS score and choroidal thickness. We divided the patients into two groups according to active and inactive disease and our groups were not equal in number.

To our knowledge, our study is the first study in the literature that evaluates choroidal thickness in Graves’ ophthalmopathy according to the presence of proptosis. Although we expected increased choroidal thickness in the proptosis group due to venous congestion or proteoglycan accumulation, we found no significant difference in choroidal thickness among the proptosis group, non-proptosis group, and the controls. This result may be associated with the spontaneous decompression effect of proptosis. Proptosis may not be a sign of increased intraorbital pressure, because proptosis can cause a decrease in intraorbital pressure and relieve venous congestion and improve circulation. The relationship between Graves’ ophthalmopathy and choroidal thickness is still unclear. Various mechanisms that are not yet fully known may play a role in choroidal thickness in patients with Graves’ disease.

## Conclusion

In this study, we found that the choroidal thickness was not associated with proptosis or clinical activation score. Only age and axial length were found to have an effect on choroidal thickness.

## Figures and Tables

**Table 1:**
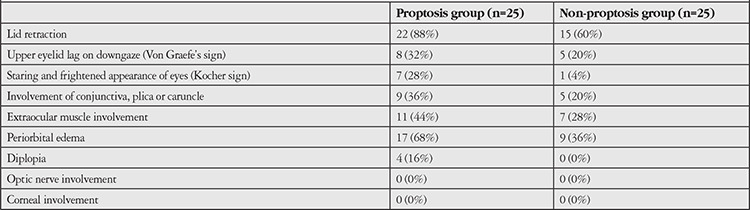
Clinical findings of the patients in the proptosis and non-proptosis group

**Table 2:**
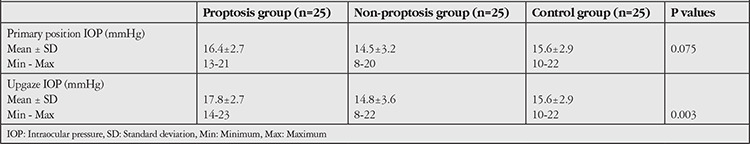
Average IOP values in primary gaze position and upgaze in the proptosis group, non-proptosis group, and control group

**Table 3:**
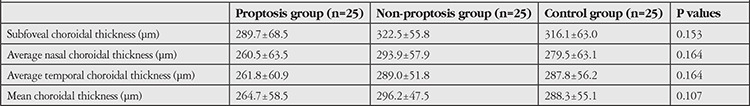
Choroidal thickness values in the proptosis group, non-proptosis group, and control group

**Table 4:**
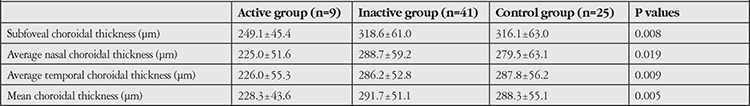
Choroidal thickness values in the clinically active, inactive, and control groups
